# *APOE* Genotype and Cardio-Respiratory Fitness Interact to Determine Adiposity in 8-Year-Old Children from the Tasmanian Infant Health Survey

**DOI:** 10.1371/journal.pone.0026679

**Published:** 2011-11-01

**Authors:** Justine A. Ellis, Anne-Louise Ponsonby, Angela Pezic, Elizabeth Williamson, Jennifer A. Cochrane, Joanne L. Dickinson, Terence Dwyer

**Affiliations:** 1 Environmental and Genetic Epidemiology Research, Murdoch Childrens Research Institute, Parkville, Australia; 2 Menzies Research Institute, University of Tasmania, Hobart, Australia; 3 Department of Physiology, University of Melbourne, Melbourne, Australia; Universite de Montreal, Canada

## Abstract

APOE plays a well established role in lipid metabolism. Animal model evidence suggests *APOE* may also be associated with adiposity, but this has not been thoroughly investigated in humans. We measured adiposity (BMI, truncal fat mass, waist circumference), physical activity (PA), cardiorespiratory fitness and *APOE* genotype (E2, E3, E4) in 292 8-year-old children from the Tasmanian Infant Health Survey (TIHS), an Australian population-based prospective birth cohort. Our aims were to examine the association of *APOE* with child adiposity, and to examine the interplay between this association and other measured factors. We found that *APOE* was associated with child lipid profiles. *APOE* was also associated with child adiposity measures. The association was E4 allele-specific, with adiposity lower in the E4-containing group (BMI: Mean difference -0.90 kg/m^2^; 95% confidence intervals (CI) -1.51, -0.28; p = 0.004). The association of *APOE4* with lower BMI differed by fitness status (difference in effect p = 0.002), and was more evident among the less fit (mean difference -1.78 kg/m^2^; 95% CI -2.74, -0.83; p<0.001). Additionally, associations between BMI and lipids were only apparent in those of lower fitness who did not carry *APOE4*. Similar overall findings were observed when truncal fat mass and waist circumference were used as alternative adiposity measures. *APOE4* and cardiorespitatory fitness could interact to influence child adiposity. In studies addressing the genetic determinants of childhood obesity, the context of child fitness should also be taken into account.

## Introduction

The plasma protein apolipoprotein E (apoE), encoded by the gene *APOE*, plays an important role in lipid metabolism. ApoE carries lipids in the bloodstream, and mediates the influx of lipids into cells such as adipocytes [1]. There are three isoforms; ε2, ε3 and ε4, encoded by three alleles, E2, E3, and E4. The isoforms differ in their binding affinities for lipids and lipoprotein receptors [2].

There is a well-established relationship between serum LDL cholesterol (LDL-C) and genotypes of *APOE* in adults and in children. The relationship is approximately linear, with E2 associated with the lowest, and E4 associated with the highest, LDL-C, as demonstrated by Bennet and colleagues by meta-analysis [2]. For children specifically, equivalent relationships between *APOE* genotype and LDL-C have also been repeatedly demonstrated [3], [4], [5], [6], [7].

The determination of body mass index (BMI) and the development of obesity are thought to be controlled, at least in part, by the regulation of lipid flux by adipose tissue [1]. Adipose tissue serves as a storage site for triglycerides (TGs) derived from lipoproteins delivered by the circulation. ApoE is highly expressed in adipocytes, and rodent *apoe* knockouts (EKO) show smaller adipocytes containing significantly less TG compared to wildtype. Whilst circulating apoE has been shown to also play a role, transplantation of EKO adipocytes into wildtype animals has demonstrated the importance of endogenous apoE in determining adipocyte size and TG content [1], [8].

There is evidence to suggest that the ability to store TGs may differ between human apoE isoforms. When fed a ‘Western-style’ high-fat diet for eight weeks, knock-in mice containing human *APOE3* gained 30% more weight than *APOE4* knock-ins [9]. Thus, human *APOE* genotype may be important in determining adiposity.

One adult study investigating *APOE* with lipids and coronary risk suggested that there may be an association between *APOE* genotype and BMI, with E4 carriers having a (non-significantly) lower BMI than E3/E3 or E2 carriers in both whites and African Americans [10]. However, recent large scale genome-wide association analyses [11] have not detected an association of *APOE* genotype with human adiposity. Little data has been available for children, however, it might be argued that child studies may provide a clearer picture due to a lack of confounding by co-morbidities and resultant medication.

Further, effect modification of the *APOE*-BMI association will be important to consider. A recent study examining interaction between obesity-associated genes and physical activity in European adults indicated that genetic predisposition to obesity is significantly attenuated by a physically active lifestyle [12]. Studies including The Cardiovascular Risk in Young Finns Study have demonstrated that physical activity (PA) appears to act as an effect modifier on the relationship between *APOE* genotype and serum lipids [7], [13]. Thus, given that PA, and also cardiorespiratory fitness (CRF), have obvious effects on energy balance, we hypothesised that PA and/or CRF might also modify any effect of *APOE* on adiposity. We therefore explored the relationship between *APOE* genotype, lipids, PA, CRF, and measures of adiposity, using relevant data available from participants of the Tasmanian Infant Health Survey (TIHS), a prospective birth cohort study.

## Materials and Methods

### Ethics Statement

The Human Research Ethics Committee (University of Tasmania) approved each stage of the study. Parental written consent and child verbal assent was obtained.

### Study sample

Singleton participants in the TIHS born in 1989 who had participated in a follow-up study in Southern Tasmania at age 7–8 years [14], [15] and were still residing in the South of the State (a defined geographical region based on telephone code) in 2002, were eligible for this study.

During the years 1988-1995, 10,562 newly-born infants were recruited into the TIHS, which had been established to investigate sudden infant death syndrome (SIDS) [16]. The selection of eligible singleton subjects was based on SIDS risk factors as described previously [17]. Parents of 1182 of the surviving infants among 1256 eligible singletons who were born specifically in 1989 participated in an in-hospital interview. In 1997, 538 of these children (now 8 years of age) who were deemed eligible for a cholesterol study were identified at schools in southern Tasmania and invited to participate; 388 did so. Detailed anthropometric measures and fasting blood samples for lipid analysis and measurement of glucose and insulin were collected [14], [15]. In 2002, 353 of these were traced to an address in southern Tasmania and 339 agreed to participate in a further study investigating genetic factors. Of these, 292 previously had skin folds and serum insulin measured at 8 years of age in 1997, and these children formed the sample for this and previous gene-environment studies [18].

### Measurements

Children underwent measurements of anthropometry and had a fasting blood sample taken. Weight was measured in light clothing using scales that were calibrated daily. Height was measured in bare feet using a stadiometer. BMI was calculated as the ratio of weight to squared height. Waist circumference was measured. Skin-fold thickness was measured with callipers at the mid-abdominal, subscapular, and suprailiac sites, with triplicate measures taken at each site and averaged. Truncal fat mass was calculated as the mean skin-fold thickness from these three sites.

Blood samples were analysed by a laboratory accredited by National Association of Testing Authorities and participating in Royal College of Pathologists of Australasia/Australasian Association of Clinical Biochemists external quality assurance programmes. The Vitros analyser was used for biochemical estimation of serum TG (Ortho Clinical Diagnostics). Child physical activity (PA) was measured by pedometer over one school lunchtime. The number of recorded steps was adjusted for the length of time the pedometer was worn. PA was also recorded via parent-reported usual activity during lunchtime [15]. Reported activities were categorised into low-moderate (sit/talk, read/study, walk, use play equipment) or high (run, sports training) activity levels for the purposes of analysis. Child CRF was assessed by the 20 metre endurance multistage shuttle run test. This test required children to run back and forth between two lines set 20 m apart with a protocol of increasing velocity and scoring by laps completed at various shuttle run velocity levels [19]. This test has been validated as a measure of aerobic fitness in children [20].

### DNA collection, extraction and genotyping

Buccal mucosa swabs were collected with Gentra PureGene brushes and DNA extracted using PureGene DNA Isolation Kits (Gentra Systems, MN, USA). The two nucleotide substitutions, a C to T at codon 112 (Cys112Arg, rs429358) and a C to T at codon 158 (Cys158Arg, rs7412) in APOE that encode the E2, E3 and E4 alleles were detected as described previously [18].

### Data analysis

Data were described using means and standard deviations or medians and inter-quartile ranges. The distribution of TG was right skewed and was log-transformed prior to analysis. Characteristics of the participants were compared between *APOE* groups using analysis of variance and chi-squared tests.

Consistent with a number of previous studies [2], *APOE* genotypes were grouped into three categories according to E2 and E4 carrier status: E2 carriers (E2/E2, E2/E3), E4 carriers (E4/E4, E4/E3), and reference (E3/E3). Individuals carrying E2/E4 (n = 7) were excluded from this classification. We then dichotomised the *APOE* genotypes in two ways: E4-containing (E3/E4, E4/E4) versus not E4-containing (E2/E2, E2/E3, E3/E3), and E2-containing (E2/E2, E2/E3) versus not E2-containing (E4/E4, E4/E3, E3/E3) groups.

The association between BMI and *APOE* was investigated using linear regression models adjusted for age and sex. Estimated differences in mean BMI with 95% confidence intervals and Wald test p-values are presented. Confounders of the association between BMI and *APOE* were identified on the basis of whether the estimated association changed by more than 10% when the confounder was entered into the model. Linear regression models of BMI on *APOE* further adjusted for lipids and insulin were fitted to separate direct effects of *APOE* on BMI from those potentially mediated through the lipids or insulin. Finally, sex, PA and CRF were each considered as potential modifiers of the association between BMI and *APOE*. Each potential modifier was added singly to the regression model with a product term representing the interaction between *APOE* and the potential modifier. PA and CRF were initially included as continuous variables, then dichotomised at the median.

The association between lipids and *APOE* was investigated using linear regression models adjusted for age and sex. CRF was considered as a potential modifier of this association, so was added to the model with an interaction term between dichotomised fitness and *APOE*.

Finally, a linear regression model of BMI on lipids was fitted within categories defined by dichotomised CRF (high fit: at or above median, vs low fit: below median) and *APOE* genotype (E4-containing vs not E4-containing), adjusted for age and sex.

P-values presented are two-sided. A p-value less than 0.05 was considered significant. All data analyses were performed in Stata Version 10.1 (Statacorp).

## Results

### *APOE* genotype is associated with lipid profile in the TIHS

The sample consisted of 292 children (208 boys, 84 girls) with a mean age of 8.2 years. A total of 290 children (99.3%) were successfully genotyped for the *APOE* variants. The genotype frequencies for the two SNPs were as follows: rs429358 TT 71.7% (208/290), TC 25.9% (75/290), CC 2.4% (7/290); rs7412 TT 1.0% (3/290), TC 14.8% (43/290), TT 84.1% (244/290). Both variants were in Hardy Weinberg Equilibrium (rs429358: p = 0.99; rs7412: p = 0.78). Initially, *APOE* genotypes were grouped into three categories according to E2 and E4 carrier status: E2 carriers (n = 39), E4 carriers (n = 75), and E3/E3 reference (n = 169). Table 1 shows characteristics of the study sample by *APOE* genotype. As expected, we found strong evidence that *APOE* genotype was associated with LDL-C (p<0.001), and to a lesser extent with HDL/Total cholesterol ratio (HDL/Total-C) and log-transformed triglyceride (TG) levels. Additionally, *APOE* genotype was associated with a number of adiposity measures, including BMI (p = 0.01), skinfold measures (eg suprailiac, p = 0.03), and waist circumference (p = 0.02). No associations were detected between *APOE* genotype and CRF or PA.

**Table 1:** Characteristics of the participants by ApoE genotype.

	E3/E3 (n = 169)		E2 containing (n = 39)		E4 containing (n = 75)		p-value§
	Mean	SD	Mean	SD	Mean	SD	
Child age	8.20	0.31	8.15	0.35	8.14	0.31	0.27
Male	73.37% (124/169)		58.97% (23/39)		26.87% (54/75)		0.20
Serum Total Cholesterol (mmol/l)	4.32	0.70	4.11	0.64	4.45	0.70	0.05
Serum LDL-cholesterol (mmol/l)l	2.63	0.64	2.26	0.50	2.75	0.66	<0.001
Serum HDL-cholesterol (mmol/l)	1.53	0.33	1.66	0.36	1.53	0.32	0.07
Serum HDL/total cholesterol ratio	0.36	0.08	0.41	0.08	0.35	0.08	0.001
Serum triglycerides (mmol/l)*	0.66	0.56, 0.87	0.81	0.64, 1.06	0.76	0.60,0.97	0.01
Birthweight (kg)	3.17	0.80	3.14	0.76	3.05	0.80	0.55
Height (cm)	128.61	6.44	129.19	5.43	126.73	6.54	0.06
Weight (kg)	28.60	5.78	29.31	5.67	26.41	4.50	0.01
Body mass index	17.18	2.52	17.45	2.46	16.34	1.65	0.01
Waist circumference (cm)	60.67	6.75	60.76	6.48	58.33	4.90	0.02
Mid-abdominal skinfold (mm)	6.09	3.80	6.78	4.20	4.66	3.60	0.20
Suprailiac skinfold (mm)	6.03	2.53	5.66	2.87	3.44	2.80	0.03
Subscapular skinfold (mm)	7.72	4.38	7.82	4.17	6.44	1.91	0.04
Truncal fat mass (mm)	8.94	5.26	9.25	5.36	7.46	3.18	0.06
Serum insulin (mU/l)	7.31	4.80	8.01	4.21	6.16	2.72	0.06
Serum fasting glucose (mm/l)	4.47	0.47	4.46	0.38	4.44	0.45	0.87
Cardiorespiratory fitness score†	18.47	8.96	18.49	8.11	17.68	7.56	0.79
Physical activity score‡	60.35	22.63	60.34	23.34	61.07	21.28	0.97

*Data presented as median and interquartile range.

† Cardiorespiratory fitness score (CRF)  =  number of laps completed in shuttle test.

‡ Physical activity score (PA)  =  number of steps/minutes pedometer worn.

§ Analysis of Variance (unadjusted). χ^2^ analysis used for sex.

### *APOE4*, but not *APOE2* is associated with adiposity

Table 2 shows the characteristics of participants after dichotomising into E4-containing (n = 75) and not E4-containing (n = 208) genotype groups. Of the lipid measures, only higher LDL-C was associated with E4-containing genotype. (p = 0.03). However, E4 was shown to be associated with reduced adiposity (eg. E4-containing: lower BMI, p = 0.004; reduced truncal fat mass, p = 0.02; lower waist circumference, p = 0.006).

**Table 2:** Lipid and body size characteristics in E4-containing and Not E4-containing*APOE* genotype groups.

	E4 containing (n = 75)		Not E4-containing (n = 208)		
	Mean	SD	Mean	SD	p-value†
Body mass index	16.34	1.65	17.23	2.50	0.004
Waist circumference (cm)	58.33	4.90	60.69	6.68	0.006
Truncal fat mass (mm)	7.46	3.18	9.00	5.27	0.02
Serum Total Cholesterol (mmol/l)	4.45	0.70	4.28	0.69	0.08
Serum LDL-cholesterol (mmol/l)l	2.75	0.66	2.56	0.63	0.03
Serum HDL-cholesterol (mmol/l)	1.53	0.32	1.56	0.34	0.58
Serum HDL/total cholesterol ratio	0.35	0.08	0.37	0.08	0.07
Serum triglycerides (mmol/l)*	0.76	0.60, 0.97	0.70	0.57, 0.92	0.37

† Linear regression (unadjusted).

*Data presented as median and interquartile range.

When participants were dichotomised into E2-containing (n = 39) and not E2-containing (n = 244) genotype groups, E2-dichotomised *APOE* genotype was associated with measures of lipids (E2-containing: lower LDL-C, p<0.001; higher HDL/Total-C ratio, p<0.001; higher HDL-C, p = 0.02; higher TG, p = 0.01), but there was no evidence of association of *APOE2* with adiposity.

### *APOE4* remains associated with BMI after adjustment for confounders

The evidence of association between *APOE4* and lower BMI was of particular interest. To further explore this, we considered the involvement of possible confounders. First, adjustment for age and sex did not alter the association (Mean difference  =  0.87 kg/m^2^; 95% CI 0.26, 1.47; p = 0.006). A number of other variables in the dataset that were potentially related to BMI were also considered as confounders, including birth weight, length and head circumference, maternal age, alcohol consumption by the mother in pregnancy, and also CRF and lower PA in childhood. None materially changed the association when added into the model individually.

### An investigation of whether lipids and insulin may be on the causal pathway from *APOE4* genotype to BMI

We tested the hypothesis that the association of *APOE4* with BMI may be party mediated by the relationship between *APOE4* and LDL-C. We found that the magnitude of the association between *APOE4* and BMI was not reduced when adjusted for LDL-C (Mean difference  =  0.99 kg/m^2^; 95% CI 0.38, 1.61; p = 0.002). *APOE4* was also associated with insulin (p = 0.027); adjustment of the *APOE4* - BMI association by insulin reduced, but did not remove, the relationship (Mean difference  =  0.75 kg/m^2^; 95% CI 0.15, 1.36; p = 0.015). Similar adjustments for other lipids and glucose also did not alter the association. Therefore, the association between *APOE4* genotype and lower child BMI appeared independent of measured metabolic profile.

### Physical activity and fitness are associated, but only fitness is associated with lipids

PA and CRF were found to be moderately associated. For example, children with a higher lunchtime pedometer score were more likely to complete extra laps of the shuttle test (p = 0.011). No associations were seen between PA (pedometer or parent-report) and lipids (adjusted for age and sex). However, higher fitness was associated with higher HDL-C (p = 0.004), higher HDL/Total-C ratio (p = 0.034), and lower TG (p = 0.026) (adjusted for age and sex). Therefore fitness appeared more relevant to lipid profile than PA in this study.

### Fitness appears to modify the relationship between *APOE4* and BMI

We considered three possible effect modifiers of the *APOE4* – lower BMI association; sex, PA, and CRF. No interactions were detected for sex (adjusted for age), or for either PA measure (adjusted for age and sex). However, a significant interaction was identified between *APOE4* and CRF (adjusted for age and sex) in relation to BMI. When assessed as a continuous variable, lower fitness potentiated the influence of *APOE4* on BMI (difference in effect (significance of interaction term) p = 0.022). When CRF was dichotomised at the median, it was found that the association between *APOE4* and lower BMI was strong in those of low fitness (n = 142) (Mean difference  =  -1.78 kg/m^2^; 95% CI -2.74, -0.83; p<0.001), but was absent in those of high fitness (n = 148) (Mean difference  =  0.074 kg/m^2^; 95% CI -0.59, 0.73; p = 0.83) (difference in effect p = 0.002) (Table 3).

**Table 3:** Interaction between *APOE* genotype and fitness in determining BMI.

			BMI (kg/m^2^)		
Fitness	*APOE*	n	Mean (SD)	Mean difference (95% CI)*	P value
High	Not E4-containing	104	16.39 (1.69)		
	E4-containing	34	16.44 (1.68)	0.074 (-0.59, 0.73)	0.825
Low	Not E4-containing	104	18.07 (2.88)		
	E4-containing	41	16.25 (1.65)	-1.78 (-2.74, -0.83)	<0.001
Difference in effect †					0.002

Fitness measured by shuttle test and dichotomised at the median.

*Linear regression adjusted for age and sex.

† Difference in the association between E4 and BMI by fitness category.

### Neither PA nor fitness appears to modify *APOE4* - lipid associations

The association between *APOE4* and higher TG was stronger among those with high parent-reported PA (difference in effect p = 0.030, adjusted for age and sex). Fitness status did not modify the association between *APOE4* (adjusted for age and sex) and Total-C (difference in effect p = 0.84), LDL-C (p = 0.64), HDL-C (p = 0.58), HDL/total-C ratio (p = 0.49), or TG (p = 0.30).

### Association between BMI and lipids is determined by fitness and *APOE4* genotype

We also examined the associations between BMI and lipids. Both HDL/total-C ratio (p = 0.028) and TG (p<0.001) were significantly associated with BMI overall. When CRF was considered as an effect modifier, it was demonstrated that these associations were only present in those of lower CRF (adjusted for age and sex) (BMI and HDL/total-C ratio p = 0.022; difference in effect p = 0.034; TG p = 0.001; difference in effect p = 0.038). This suggests that the apparent lack of effect of *APOE4* on BMI that is evident in children with higher fitness may be related to an uncoupling of the relationship between BMI and blood lipids and with greater fitness.

Further, when the fitness groups were then dichotomised by presence or absence of *APOE4*, it was found that significant associations between BMI and lipids were only evident in those who were non-E4 and lower fitness (Table 4). It is in this same group that BMI is significantly elevated when compared to all other fitness/*APOE4* combinations (Table 3). Thus, presence of an *APOE4* allele appears to provide protection from increased BMI in children of low CRF, by removing the BMI-lipid association similarly to that found in children of higher fitness (and lower BMI). In contrast, low-fit non-E4 children tended to have higher BMI, and a strong relationship between BMI and lipid levels, especially a positive relationship between BMI and TGs.

**Table 4:** Associations between BMI and blood lipids in the low fitness group by absence (n = 104) or presence (n = 41) of an *APOE4* allele.

	No *APOE4* allele		*APOE4* allele	
Lipid	Regression coefficient (95% CI)*	P value	Regression coefficient (95% CI)*	P value
Total Cholesterol	0.60 (-0.19, 1.39)	0.133	-0.04 (-1.01, 0.93)	0.94
LDL-C	1.06 (0.15, 1.98)	0.023	0.22 (-0.58, 1.03)	0.58
HDL-C	-1.47 (-3.20, 0.27)	0.097	-0.68 (-2.23, 0.85)	0.37
HDL/Total-C ratio	-11.38 (-19.08, -3.69)	0.004	-3.46 (-10.25, 3.33)	0.31
Log triglycerides	2.55 (1.03, 4.08)	0.001	1.03 (-0.58, 2.63)	0.20

Fitness measured by shuttle test and dichotomised at the median. There were no associations between BMI and blood lipids for either of the high-fit-*APOE4* genotype combinations.

*Adjusted for age and sex.

### Serum insulin

Given that insulin resistance and obesity appear intrinsically linked, we performed further analyses to consider the role of insulin in the identified associations and interactions. None of the observed relationships were materially altered by adjustment for insulin. Particularly, the association of *APOE4* and BMI was still evident only in the low-fit group following adjustment for insulin (low-fit p = 0.001; high-fit p = 0.71). Therefore, the effect of *APOE4* and fitness on BMI appeared independent of mechanisms related to serum insulin.

### Similar associations are seen for other measures of adiposity

We performed similar analyses on truncal fat mass and waist circumference, including association with *APOE4*, adjustment for confounders, and interaction with CRF, and observed similar associations (data not shown). Therefore, these similar relationships demonstrate consistency in the pattern of findings not only for BMI but across various measures of adiposity.

## Discussion

This cohort sample incorporates a rare combination of childhood measures, including adiposity, *APOE* genotype, blood lipid profile, and PA and CRF. Using this study, we have identified that *APOE4* appears to be associated with reduced adiposity in children. Additionally, this effect is modified by CRF; in children of lower CRF, *APOE4* seems to predispose to lower adiposity, but also to a more atherogenic serum lipid profile. These findings show the importance of examining *APOE*, adiposity and blood lipids in the context of related PA and fitness.

There is a well-established association between *APOE* genotype and blood lipids in both adults and children [2] [3], [4], [5], [6], [7]. In the TIHS, as expected, *APOE4*-carrying individuals had significantly higher LDL-C levels than those carrying other alleles. We also observed an association between *APOE* genotype and adiposity. Individuals with an *APOE4*-containing genotype had lower mean BMI, truncal fat mass and waist circumference than those with other genotypes. This suggests that the E4 allele may have a protective effect against increased adiposity. Little evidence for a conversely detrimental effect of E2 on adiposity was observed.

Insight into the possible mechanism(s) through which *APOE* regulates fatness might be gleaned from consideration of the regulation of lipid flux by adipocytes. Adipocytes act as storage compartments for TGs. TGs are either produced endogenously by adipocytes from internalised free fatty acids that are the product of triglyceride rich lipoprotein (TGRL) breakdown in the circulation, or from internalised TGRLs themselves, which are broken down endogenously. In the circulation, ApoE carries TGRLs, such as VLDLs and chylomicrons, to adipose cells, and thus ApoE inefficiency has the capacity to reduce clearance of lipid from the circulation into adipose tissue. EKO mice demonstrate smaller adipocytes, and less adipose tissue [21]. Recent studies have demonstrated that endogenous ApoE modulates the TG content of adipocytes, independently of exogenous ApoE [1], [8]. Further, when mice with either knocked-in human *APOE3*, or *APOE4*, were subjected to high fat diets for 8 weeks, E4 knock-ins were significantly more resistant to obesity than E3 knock-ins [9]. Taken together, these data suggest that a possible mechanism through which E4 may protect against adiposity is via a reduction in the ability of adipocytes to accumulate TG.

PA was recently demonstrated to modify the effect of obesity related genes in adults [12]. PA has also been shown to modify the effect of *APOE* genotype on lipids, particularly LDL-C, in children and adults [7], [13]. We saw little evidence of such an interaction when considering both an objective, and a subjective measure of PA, although the ability to fully test this was limited by the fact that we collected objective PA data at only one time-point in a single day. However, a novel aspect of this study was our ability to also examine the effect of CRF on the child *APOE*-adiposity association. This is of relevance because CRF appears more important in controlling cardiovascular disease risk factors than PA [22]. Our data concurs with recent work demonstrating that the association between CRF and PA is generally weak [23], especially in children [24]. It is likely that CRF is determined not only by a number of environmental factors that includes PA, but also by genetic factors [25]. Thus there is a clear need to consider PA and CRF separately. We found no interaction between PA and *APOE* genotype in determining BMI. However, we did find that *APOE4* was strongly associated with lower BMI only in those of lower CRF, and had little effect on BMI in those of higher CRF. This suggests that the mode of action through which *APOE4* exerts its apparent protective effect against adiposity may only be relevant in those who are less fit.

We also examined the relationship between blood lipids and BMI in children grouped by *APOE* variant and fitness. There was little association between lipids and BMI, except in the low-fit, non-E4 allele group, where BMI was associated with TG, LDL-C, and HDL/Total-C ratio. We suggest that in children of higher fitness, lipid may be more efficiently removed from circulation via, for example, muscle metabolism, and in those carrying *APOE4*, storage of lipid by adipocytes is reduced. Only in children who are of lower fitness and who lack *APOE4* may be free to take up and store lipids at a rate that is mainly dependent on circulating lipid levels.

The relationship between *APOE4*, circulating LDL-C, cardiovascular risk, and BMI warrants further scrutiny. It is well established that *APOE4* predisposes to higher circulating LDL-C, along with higher cardiovascular risk [2]. In the TIHS, we confirmed the association of E4 with higher LDL-C, however the study design precluded assessment of cardiovascular risk. The lower BMI seen in E4 carriers appears somewhat counterintuitive to higher cardiovascular risk that might be predicted to be present in these individuals. But the existence of a direct relationship between high BMI, as one of a web of metabolic syndrome factors, and increased cardiovascular risk, is unclear [26], [27], [28], [29]. Barriers to the deposition of lipid into adipocytes may result in lower BMI, but may also drive higher circulating levels of lipids such as LDL-C and thus a more detrimental atherogenic lipid profile and higher cardiovascular risk. Thus, it is possible that while *APOE4* might confer protection from fatness, it may also confer higher risk of cardiovascular disease.

It is also pertinent to mention that, despite recent large scale genome-wide association analyses [11], association of *APOE* genotype with human adiposity has not, to our knowledge, been reported previously. We suggest that this may be because genome-wide association studies have by-and-large focussed on adult obesity. We also suggest that the effects of *APOE* on adiposity may not be strongly and replicably apparent without both specific consideration of the E4 allele, and the interaction of E4 with CRF. Additionally, the lack of consistency of association of *APOE* genotype with various lipids such as TGs and HDL-C across numerous studies [2], [30], [31], [32], [33] may be due to the interacting effect of BMI and CRF, the latter of which is rarely taken into consideration in such analyses. Our findings require independent confirmation in cohorts where data on both *APOE* genotype and CRF is available. However, we suggest our data demonstrates the importance of the consideration of environmental factors in tandem with genetic factors, in order to continue to progress our understanding of the architecture of complex diseases and phenotypes [34].

It is interesting to speculate as to whether *APOE* may be a ‘thrifty’ gene during times of food abundance. Thrifty genes are historically advantageous in times of famine, but are rendered detrimental by modern high fat diets and sedentary lifestyles [35]. In Caucasians, as in most populations, the *APOE*3 allele is the most frequent, and is in high frequency in the non-*APOE4*-containing genotype carriers to which we compared our E4-containing carriers. Our data suggests that E3 (and also possibly E2) may provide more efficient fat deposition than E4, and may be the thrifty allele(s) that in the face of high fat diets and low fitness levels, now predispose to higher BMI. The modern *APOE4* allele might protect against fat deposition, however it appears to also predispose to a more atherogenic lipid profile [2], thus leading to a higher risk of cardiovascular disease in *APOE4* carriers.

In conclusion, in 8 yr old children from the TIHS, *APOE4* is associated with lower BMI. The relationship between *APOE4* and lower BMI is potentiated by lower CRF. We propose that, in those of lower CRF, *APOE4* compensates for the lack of the protective effect of fitness from increased adiposity. Based on animal model studies, the mechanism through which *APOE4* may protect against increased adiposity may involve a decreased ability of E4-expressing adipocytes to take up, synthesise, and/or store TGs. At a future clinical level, we suggest that assessment of child CRF and *APOE4* genotype may potentially define a subgroup that is more vulnerable to increased adiposity, and therefore more at risk of later obesity-related disease. Although our sample size is relatively small, our data provide strong impetus for further examination of the role of *APOE* in determining adiposity in larger studies of both children and adults.
